# Supplementary material for: Representations of imaginary scenes and their properties in cortical alpha activity
Source: Sci Rep. 2024 Jun 4;14:12796. doi: 10.1038/s41598-024-63320-4 (PMC11150249; doi:10.1038/s41598-024-63320-4)
Supplement: Supplementary file 1 — Supplementary Information. [file 41598_2024_63320_MOESM1_ESM.pdf]

Representations of imaginary scenes and their properties in cortical alpha activity

Rico Stecher[1,*], Daniel Kaiser[1,2]

[1]Mathematical Institute, Department of Mathematics and Computer Science, Physics, Geography, Justus-Liebig-University Gießen, Germany

[2]Center for Mind, Brain and Behavior (CMBB), Philipps-University Marburg and Justus-Liebig-University Gießen, Germany

Supplementary Information

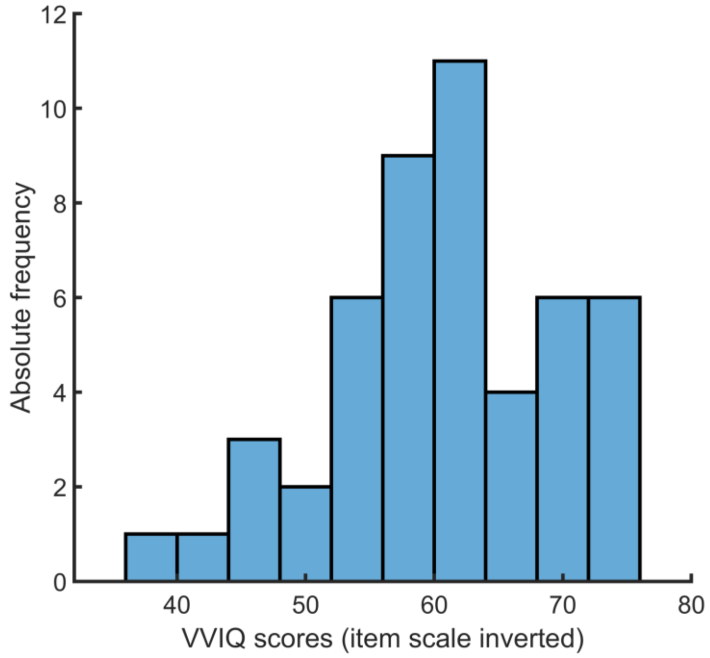

Fig S1. Histogram of the VVIQ scores of the participants.

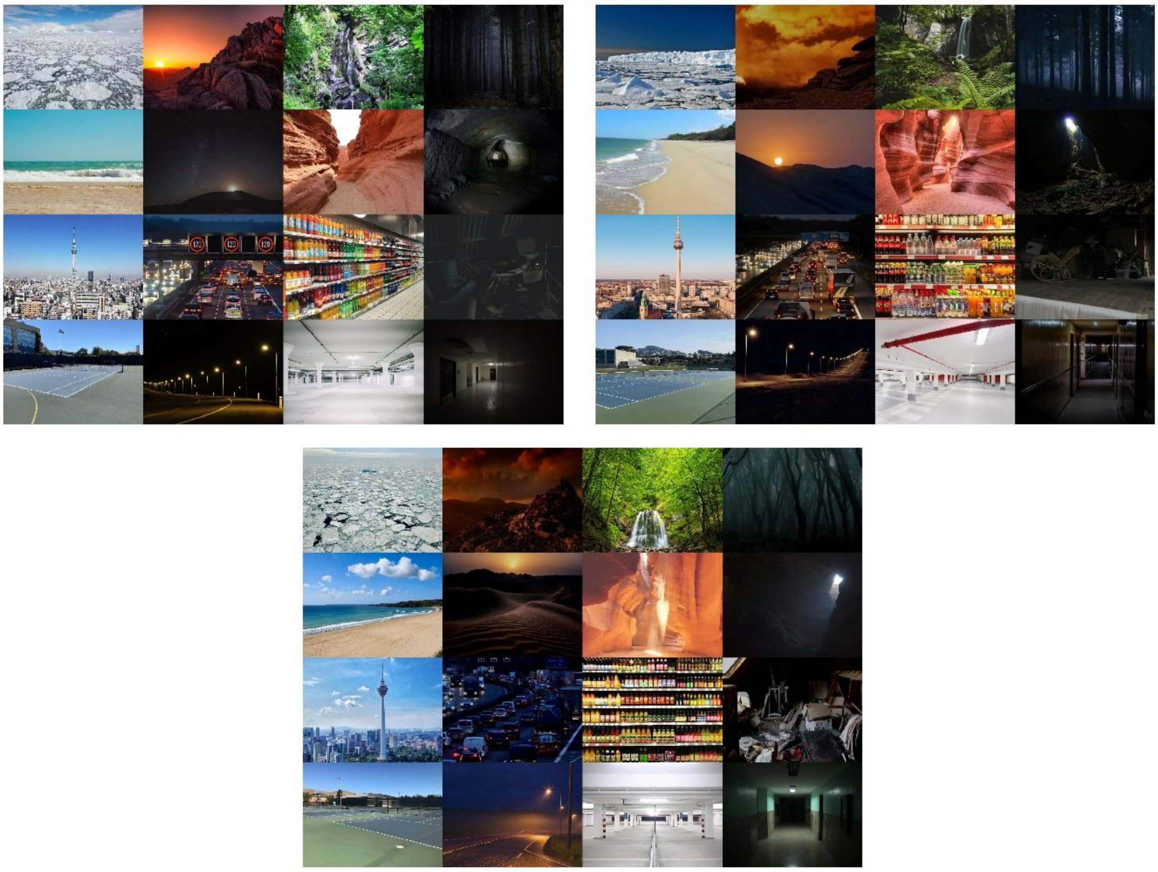

Figure S2. Stimuli used in the perception task.

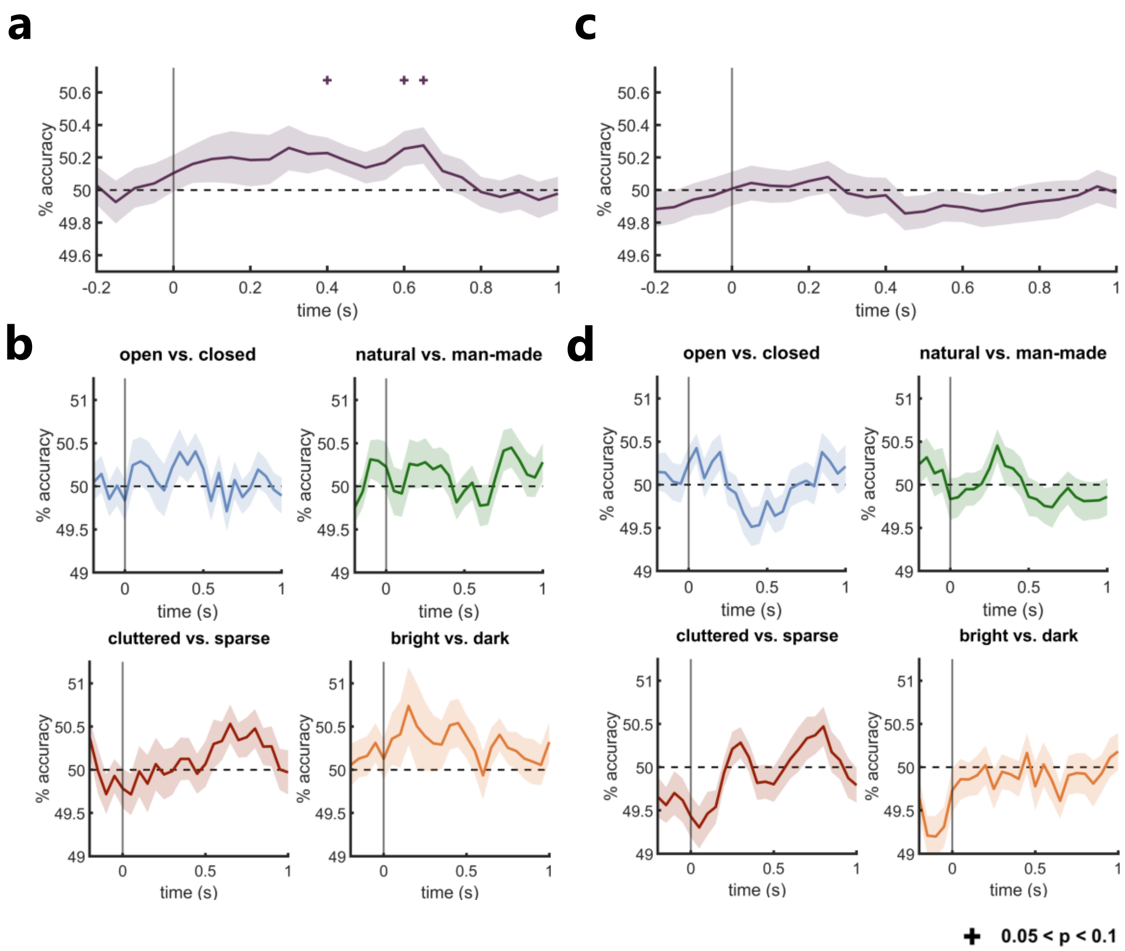

Figure S3. Imagery-perception cross-decoding in the theta and beta frequency bands. a) Mean pairwise scene cross-decoding in the theta band. b) Scene property cross-decoding in the theta band. c) Mean pairwise scene cross-decoding in the beta band. d) Scene property cross-decoding in the beta band. Apart from a marginally significant trend in the theta frequency band for the mean pairwise scene decoding, we found no significant above-chance cross-decoding performance. Error margins reflect the standard error of the mean. Cross markers indicate marginal significance at $p<0.1$ (corrected for multiple comparisons).

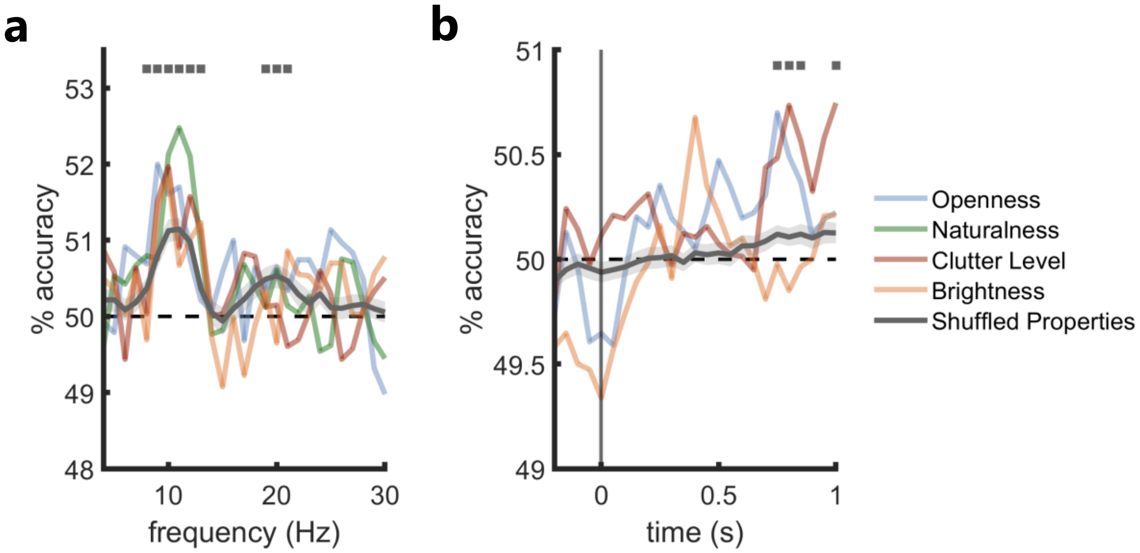

Figure S4. Shuffled property decoding across frequencies and time. a) Scene property decoding with randomized property category assignment (with the original property decoding as a reference) at each frequency from 4-30 Hz during imagery. b) Imagery-perception scene property cross-decoding in the alpha band with randomized property category assignment (with the original property cross-decoding as a reference) at each time point during perception. This analysis revealed late shared alpha band representations with imagery from around 750-850 ms and at 1000 ms during perception. These timings overlap with the marginally significant time points identified in the original mean pairwise scene cross-decoding (see Fig. 3c). Overall, both shuffled property (cross-)decoding analyses show a decoding accuracy profile that is highly similar to the mean pairwise scene (cross-)decoding (see Fig. 3a and 3c), suggesting that the shuffled property (cross-)decoding is indeed based on individual scene features. In addition, the magnitude of the accuracies is generally lower than the peak property (cross-)decoding across frequencies and time, making it seem unlikely that our shuffled property (cross-)decoding accuracies merely happened to be lower at the frequencies/time points at which we conducted the shuffled property control analysis (Fig. 4). Error margins reflect the standard error of the mean. Square markers indicate that the shuffled property (cross-)decoding significantly exceeded chance level at $p<0.05$ (corrected for multiple comparisons).

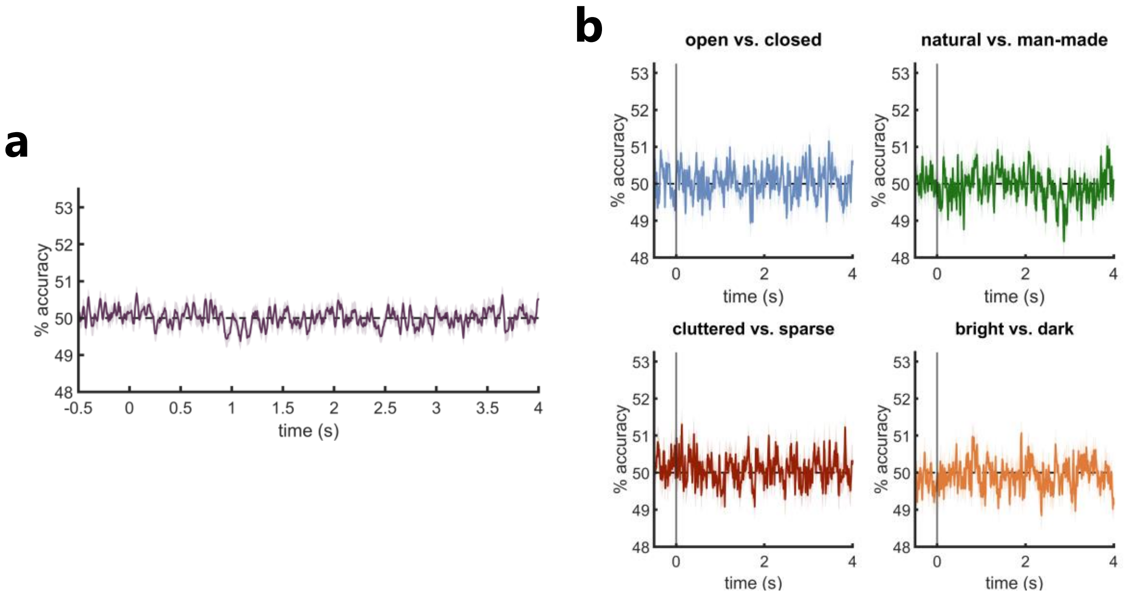

Figure S5. Time-resolved decoding of individual imagined scenes and their properties from broadband EEG responses across all channels. a) Mean pairwise scene decoding. b) Scene property decoding. Imagined scenes and their properties were not discriminable from broadband responses across time. Individual-participant time courses (200 Hz temporal resolution) were smoothed with a 5-time-point (25 ms) rolling average before averaging across participants. Error margins reflect the standard error of the mean.

Openness	Naturalness	Clutter Level	Brightness	Descriptions (German original)	Descriptions (English)
Open	Natural	Cluttered	Bright	Stell dir vor, du bist auf einem weiten Eismeer, das sich unter einem blauen Himmel bis zum Horizont erstreckt. Dein Blick schweift über einen Ozean, der mit so vielen unterschiedlichen Eisschollen bedeckt ist, dass man das Wasser kaum sehen kann. Eine unendliche Zahl weißer, irregulärer Figuren, die von kleinen Eisbrocken bis hin zu großen Schollen reichen, tummeln sich im kalten Nass.	Imagine you are on a vast arctic sea that stretches until the horizon underneath a blue sky. Your eyes survey an ocean that is filled with so many different ice floes that you can barely see the water. An infinite number of white, irregular figures, ranging from small ice chunks to large floes, are frolicking in the cold water.
Closed	Natural	Cluttered	Bright	Stell dir vor, du bist tagsüber in einem Laubwald. Du stehst vor einer hohen Felswand, von der ein Wasserfall auf eine Ansammlung zerklüfteter Steine vor dir herabstürzt. Du bist von so vielen Gräsern, Büschen und Bäumen umgeben, dass du den Himmel kaum sehen kannst, jedoch ist der Wald durch das viele Tageslicht, das durch das Blätterdach fällt, sehr erhellt.	Imagine you are in a deciduous forest during daytime. You are standing in front of a tall rock face from which a waterfall gushes down onto a small group of ragged rocks. You are surrounded by so many weeds, bushes and trees that you can barely see the sky, but, due to the intense daylight shining through the canopy, the forest is quite bright.
Open	Man-Made	Cluttered	Bright	Stell dir vor, du stehst auf einem Bürogebäude und schaust auf eine gigantische urbane Metropole. Du bist konfrontiert mit	Imagine you are standing on top of an office building, looking at a gigantic urban metropolis. You are confronted with an endless sea

				einem endlosen Meer von Wohngebäuden und Bürokomplexen unterschiedlichster Größen und Formen. Ein riesiger, schlanker Fernsehturm thront im klaren Morgenhimmel über der Szenerie.	of apartment buildings and office complexes of different sizes and shapes. A gigantic, slim TV tower lingers in the clear morning sky above the scenery.
Closed	Man-Made	Cluttered	Bright	Stell dir vor, du bist in der Getränkeabteilung eines Supermarkts. Im grellen Neonlicht schaust du auf ein Regal, das von oben bis unten mit Flaschen gefüllt ist. Die Flaschen verschiedener Marken haben eine große Palette an Farbtönen und Formen und ihre Vielzahl an Etiketten weisen auf die Getränke hin, mit denen sie gefüllt sind.	Imagine you are in the beverage section of a supermarket. You are looking at a shelf in the dazzling neon light that is filled with bottles from top to bottom. The bottles of different brands feature a great palette of colors and shapes and their myriad of labels indicate the beverages they contain.
Open	Natural	Sparse	Bright	Stell dir vor, du bist am Strand. Du bist erstaunt, dass der ebene, feinkörnige Sandstrand komplett frei von Unrat ist und dass sich keine Menschenseele hier befindet. Deine Augen ruhen auf einem sanften, blau-grünen Meer in der glühenden Nachmittagssonne, von dem vereinzelt kleine Wellen über den hellen Sand gespült werden.	Imagine you are at the beach. You are surprised, that the even, fine-grained sand beach is completely free of trash and that there is not a single person in sight. Your eyes rest upon a docile, blue-green sea in the glowing afternoon sun, from which every now and then small waves are being washed upon the bright sand.
Closed	Natural	Sparse	Bright	Stell dir vor, du bist in einer engen Schlucht zwischen zwei Klippen aus rötlichem Gestein. Obwohl die Passage	Imagine you are in a narrow canyon between two cliffs made of red stone. Even though the passage is so narrow

				so eng ist, dass du kaum den Nachmittagshimmel sehen kannst, ist deine Umgebung durch die Sonnenstrahlen sehr gut beleuchtet. Du stehst auf einem brachen, sandigen Boden, der komplett frei von Geröll ist.	that you can barely see the afternoon sky, your environment is very well lit by the sunrays. You stand on a barren, sandy ground that is completely free of rubble.
Open	Man-Made	Sparse	Bright	Stell dir vor, du bist auf einer leeren Tennisanlage. Die Anlage hat blaue Kunststoffplätze, die von dunklen Netzen überspannt werden. Obwohl die Anlage von einem schwarzen Zaun umrandet wird, kann man dahinter gut den strahlend blauen Himmel sehen, der auf der linken Seite von einem kargen Betongebäude verdeckt wird.	Imagine you are on an empty tennis facility. The facility has blue hard courts, that are spanned by dark nets. Even though the facility is surrounded by a black fence, one can see the clear blue sky that is covered by a barren concrete building on the left very well.
Closed	Man-Made	Sparse	Bright	Stell dir vor, du bist in einer Tiefgarage. Im grellen Licht der Neonröhren an der Decke siehst du, dass die Decke der Tiefgarage komplett weiß gestrichen ist und von Reihen von Säulen getragen wird. Du stellst außerdem fest, dass die Tiefgarage komplett leer ist und sich hier weder Fahrzeuge noch Personen befinden.	Imagine you are in an underground parking house. In the dazzling light of the neon tubes on the ceiling you can see that the ceiling is painted entirely white and supported by rows of pillars. You notice in addition, that the parking house is completely empty and neither vehicles nor people are to be found.
Open	Natural	Cluttered	Dark	Stell dir vor, du befindest dich auf einem Bergrücken. Zu deiner Rechten türmt sich ein riesiger Haufen bizarr zerklüfteter Felsen auf, der sich	Imagine you are on a mountain ridge. A gigantic pile of bizarre jagged rocks that stretches across almost the entire ridge towers to your right. To your left,

				über fast den ganzen Bergrücken verteilt. Zu deiner Linken siehst du die schwache Abendsonne, die gerade hinter dem Horizont verschwindet und die Szene in ein dunkles Rot taucht.	you can see the weak evening sun disappear behind the horizon, casting the scenery in a dark red.
Closed	Natural	Cluttered	Dark	Stell dir vor, du befindest dich nachts in einem düsteren Nadelwald. Im schwachen Mondlicht kannst du ausmachen, dass du völlig von einem undurchdringbaren Gewirr aus trockenen Ästen, Baumstämmen und losen Zweigen umschlossen bist. Außerdem umringt dich ein dichter Nebel, der es unmöglich macht zu erkennen, was vor dir liegt.	Imagine you are in a dark forest at night. In the faint moonlight you can make out that you are fully surrounded by an impenetrable tangle of dry branches, tree trunks and loose twigs. You are also enveloped by a thick fog that makes it impossible for you to see what is ahead of you.
Open	Man-made	Cluttered	Dark	Stell dir vor, du bist spät abends auf einer Autobahnbrücke und schaust auf einen Stau. Ein endloser Strom aus dicht an dicht aneinandergereihten PKWs und LKWs fließt zähflüssig die Fahrbahn entlang. Direkt vor dir zieht sich eine Überbrückung über die Autobahn, an der mehrere Verkehrsschilder angebracht sind.	Imagine you are on a highway bridge in the late evening, looking down upon a traffic jam. An endless stream of densely packed cars and trucks flows slowly along the road. There is a bridge crossing the motor way right in front of you on which multiple street signs are attached.
Closed	Man-Made	Cluttered	Dark	Stell dir vor, du bist in einem engen, alten Dachboden. Der Dachboden ist so dunkel, dass deine Umgebung	Imagine you are in an old and narrow attic. The attic is so dark that your environment is hard to make out. The few

				schwer auszumachen ist. Durch die wenigen Lichtstrahlen, die von außen hineindringen, kannst du sehen, dass um dich herum haufenweise Schrott liegt, darunter alte Werkzeuge, Möbel und Boxen.	light beams filtering through from outside enable you to see that you are surrounded by piles of junk, consisting of old tools, furniture and boxes among other things.
Open	Natural	Sparse	Dark	Stell dir vor, du bist in einer Wüste bei Sonnenuntergang. Du siehst der Abendsonne dabei zu, wie sie hinter einer riesigen Düne aus feinstem Sand verschwindet und die komplette Wüste in ein tiefes Schwarz taucht. In dieser beinahe vollkommenen Dunkelheit kannst du nichts und niemanden um dich herum erkennen.	Imagine you are in a desert at dusk. You are watching the evening sun disappear behind a giant dune made of the finest sand, dyeing the entire desert a deep black. In this almost complete darkness, you cannot make out anyone or anything around you.
Closed	Natural	Sparse	Dark	Stell dir vor, du bist in einer dunklen Höhle. Im Licht deiner schwachen Taschenlampe siehst du, dass die Höhle sehr eng und leer ist und ihre Wände aus blau-grünem Gestein bestehen. Du stellst zudem fest, dass vor dir ein Engpass ist, hinter dem sich der Ausgang der Höhle befindet, durch den ein winziger, entfernter Lichtkegel hereintritt.	Imagine you are in a dark cave. In the light of your weak flashlight, you can make out that the cave is very narrow and empty and that its walls consist of blue-green stone. You also notice that there is a narrow passage behind which lies the exit of the cave, from which a tiny, distant cone of light seeps in.
Open	Man-made	Sparse	Dark	Stell dir vor, du bist auf einer Landstraße. Es ist spät in der Nacht und du kannst nichts außer die Straße	Imagine you are on a country road. It’s late at night and you cannot recognize anything except for the road that slithers

				erkennen, die sich endlos bis zum Horizont schlängelt und von einer Reihe von Straßenlampen in ein sanftes, warmes Gelb gehüllt wird. Aufgrund der späten Uhrzeit befinden sich keine Fahrzeuge auf der Fahrbahn.	endlessly towards the horizon and is shrouded in a soft and warm yellow. Because it is late, there are no vehicles on the road.
Closed	Man-Made	Sparse	Dark	Stell dir vor, du bist in einem Korridor eines Bürogebäudes nachdem der Strom ausgefallen ist. Die letzte verbliebene Deckenlampe wirft einen schwachen Lichtkegel auf den Boden vor dir. Du bemerkst, dass der Korridor vollkommen leer ist und sich um dich herum keine Menschenseele befindet.	Imagine you are in the corridor of an office building after a power outage. The last remaining ceiling lamp casts a weak cone of light on the floor in front of you. You notice that the corridor is totally empty and there is not a single soul around you.

Table S1. Scene descriptions with scene property categories and English translation.
